# One Pot Single Step Synthesis and Biological Evaluation of Some Novel *Bis*(1,3,4-thiadiazole) Derivatives as Potential Cytotoxic Agents

**DOI:** 10.3390/molecules21111532

**Published:** 2016-11-15

**Authors:** Sobhi M. Gomha, Nabila A. Kheder, Abdou O. Abdelhamid, Yahia N. Mabkhot

**Affiliations:** 1 Department of Chemistry, Faculty of Science, Cairo University, Giza 12613, Egypt; s.m.gomha@gmail.com (S.M.G.); nabila.abdelshafy@gmail.com (N.A.K.); abdelhamid45@gmail.com (A.O.A.); 2 Department of Pharmaceutical Chemistry, Faculty of Pharmacy, King Khalid University, Abha 61441, Saudi Arabia; 3 Department of Chemistry, College of Science, King Saud University, P.O. Box 2455, Riyadh 11451, Saudi Arabia

**Keywords:** *bis-*hydrazonoyl chlorides, *bis*(1,3,4-thiadiazole), anticancer activity, methyl arylidene dithiocarbamate, dipolar cycloaddition reaction

## Abstract

A novel series of *bis*(1,3,4-thiadiazole) derivatives were synthesized in one step methodology with good yields by condensation reaction between *bis*-hydrazonoyl chloride **1** and various reagents. The structures of the prepared compounds were confirmed by spectral data (IR, NMR, and MS), and elemental analysis. The anticancer activity against human breast carcinoma (MCF-7) cancer cell lines was evaluated in MTT assay. The results revealed that the *bis*-thiadiazole derivatives **5c**,**d**, **7b**,**c** and **9c** had higher antitumor activity than the standard drug Imatinib.

## 1. Introduction

**Figure 1:**
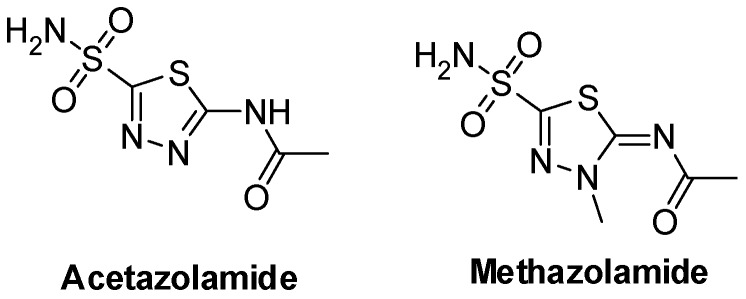
Examples of medications containing 1,3,4-thiadiazole ring.

Many compounds having a 1,3,4-thiadiazole ring in their skeleton are capable of versatile pharmacological activities [1]. Also, thiadiazoles have been introduced as anticonvulsant [2], anti-parkinsonism [3], anti-histaminic [4] and anti-asthmatic [5], antitumor [6,7], analgesic [8], antimicrobial [9,10], and antitubercular [11,12,13] agents. Additionally, many drugs containing a 1,3,4-thiadiazole ring are available in the market, such as the carbonic anhydrase inhibitors Acetazolamide and Methazolamide (Figure 1).

**Figure 2:**
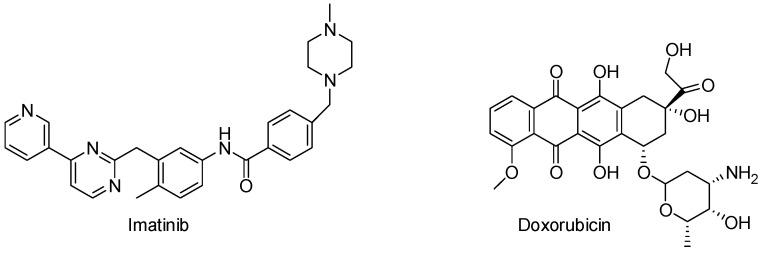
Structure of Imatinib and Doxorubicin.

Imatinib (Figure 2) is a tyrosine-kinase inhibitor used to treat chronic myelogenous leukemia, gastrointestinal stromal tumors and a number of other malignancies. Doxorubicin (Figure 2) is used to treat some leukemias and Hodgkin’s lymphoma, cancers of the bladder, breast, stomach, lung, ovaries, thyroid, soft tissue sarcoma, and multiple myeloma. In view of the medicinal importance of thiadiazole ring and as a continuation of our interest in the synthesis of a variety of thiadiazole derivatives for biological evaluation [14,15,16,17,18,19,20,21,22,23], we reported in the present study, the synthesis of a series of nine *bis*(1,3,4-thiadiazole) derivatives and their evaluation against breast cancer cell line (MCF-7) using Imatinib and Doxorubicin as a reference drugs.

## 2. Results and Discussion

### 2.1. Chemistry

**Scheme 1:**
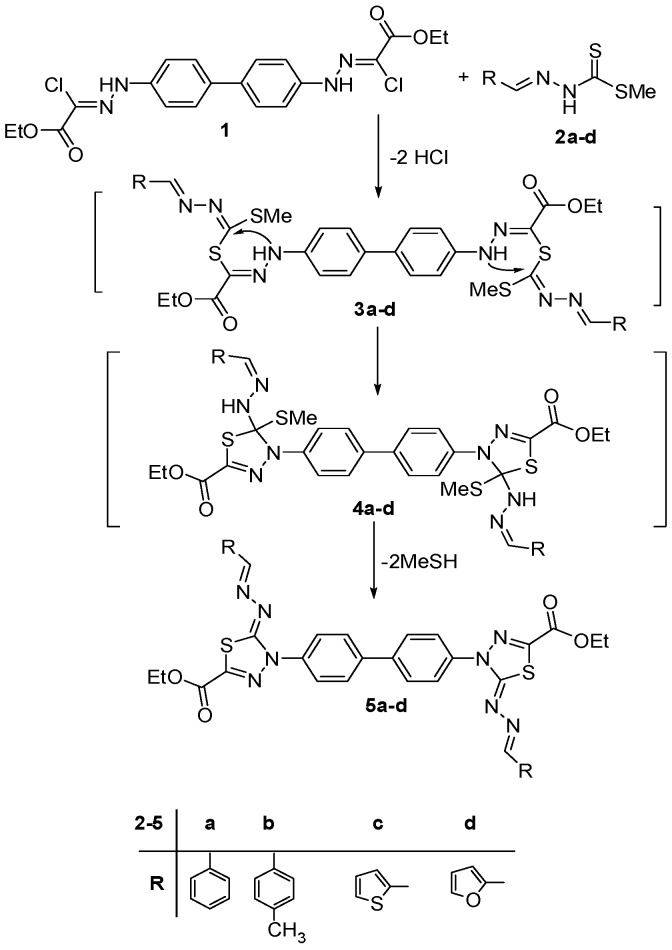
Synthesis of *bis*-thiadiazole derivatives **5a**–**d**.

A mixture of *bis*-hydrazonoyl chloride **1** [24] and the appropriate methyl arylidene dithiocarbamate **2** [25,26,27,28] was stirred at room temperature for 30 min to afford the corresponding *bis*(1,3,4-thiadiazole) derivative **5** (Scheme 1). These reactions were assumed to start in each case, via *S*-alkylation with the elimination of two hydrochloric molecules, to afford the non-isolable *bis*-alkylated intermediate **3**, which underwent intramolecular Michael-type addition to give intermediate **4**. Elimination of two methanethiol molecules from **4** gave the final product **5**. The structures of the synthesized products **5a**–**d** were elucidated based on their spectral (IR, MS, ^1^H- and ^13^C-NMR) and elemental analyses Their IR spectra showed, in each case, an absorption band in the region 1708–1733 cm^−1^ due to the carbonyl of the ester group. Also, their ^1^H-NMR spectrum exhibited a triplet signals in the region δ 1.29–1.32 and a quartet signals in the region δ 4.34–4.35 due to CH_2_ and CH_2_ protons of the ethoxy groups, in addition to the aromatic signals. The mass spectra of the compounds **5a**–**d** showed, in each case, a peak due to their molecular ions.

**Scheme 2:**
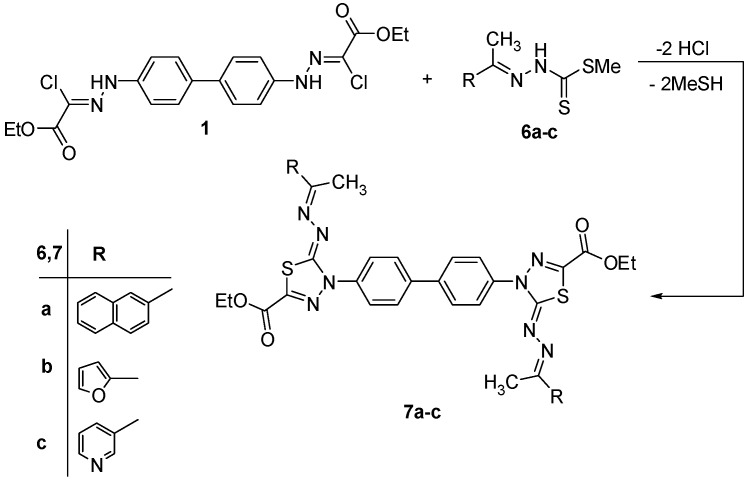
Synthesis of *bis*-thiadiazole derivatives **7a**–**c**.

**Scheme 3:**
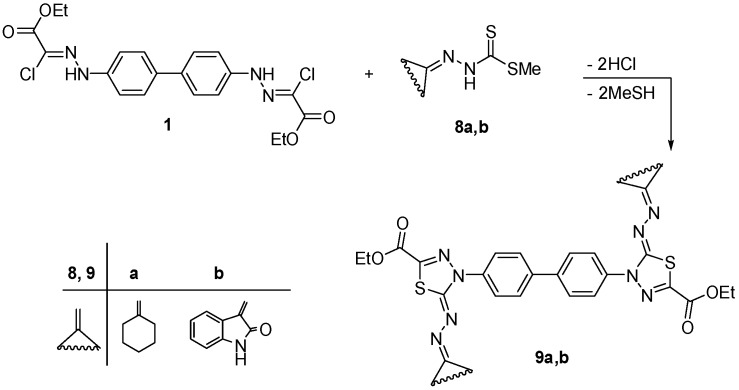
Synthesis of bis-thiadiazole derivatives **9a**,**b**.

Also, the synthesis of combinatorial libraries of *bis*(1,3,4-thiadiazole) derivatives permits the testing of the biological activities of a vast array of these compounds. So, we intended to repeat the latter experiment again using *bis*-hydrazonoyl chloride **1** with the appropriate methyl arylidene dithiocarbamate **6a**–**c** and **8a**,**b**, under the same experimental conditions, which led to the corresponding *bis*-thiadiazoles **7a**–**c** and **9a**,**b**, respectively (Scheme 2 and Scheme 3). The structures of these products **7a**–**c** and **9a**,**b** were verified by their spectral and elemental analysis.

### 2.2. Pharmacology

#### Biological Screening of the Synthesized *Bis*-thiadiazoles for Their Cytotoxic Activity

The in vitro growth inhibitory rates (%) and inhibitory growth activity (as measured by IC_50_) of the newly synthesized *bis*-thiadiazoles were determined against human breast carcinoma cell line (MCF-7) in comparison with the well-known anticancer standard drugs doxorubicin and Imatinib (Gleevec^®^), using MTT viability assay. Data generated were used to plot a dose-response curve with which the concentration (μM) of test compounds required to kill 50% of the cell population (IC_50_) was determined. Cytotoxic activity was expressed as the mean IC_50_ of three independent experiments. The difference between inhibitory activities of all *bis*-thiadiazoles with different concentrations was statistically significant *p* < 0.001. Table 1 shows the antitumor activities of the tested *bis*-thiadiazoles compared with reference standard drugs evaluated using MTT assay on breast cancer cell line (MCF-7).

The previous results lead to the following conclusions.

- The *bis*-thiadiazole derivatives **5c**,**d**, **7b**,**c** and **9b** had higher antitumor activity than the standard drug Imatinib.
- The *bis*-thiadiazole derivatives **5a**,**b** and **9a** have moderate activity.
- The *bis*-thiadiazole derivative **7a** has poor antitumor activity against human breast carcinoma cell line (MCF-7).
- The heterocyclic rings such as pyridine in **7c**, thiophene in **5c***,* furan in **5d**, **7b** and indole in **9b** are necessary to have the higher antitumor activity.

## 3. Materials and Methods

### 3.1. Chemistry

#### 3.1.1. General

Measurements of the melting points were carried out on Electrothermal IA 9000 series digital melting point apparatus (Bibby Sci. Lim. Stone, Staffordshire, UK). The IR spectra were recorded in potassium bromide discs on a Pye Unicam SP 3300 and Shimadzu FT-IR 8101 PC infrared spectrophotometer (Shimadzu, Tokyo, Japan). ^1^H-NMR and ^13^C-NMR spectra were measured in deuterated dimethyl sulfoxide (DMSO-*d*_6_) using a Varian Gemini 300 NMR spectrometer (Varian, Inc., Karlsruhe, Germany). Mass spectra were recorded on a Shimadzu GCMS-QP1000 EX mass spectrometer (Tokyo, Japan) at 70 eV. Measurements of the elemental analysis were carried out at the Microanalytical Centre of Cairo University, Giza, Egypt. All reactions were followed by TLC (Silica gel, Merck, Kenilworth, NJ, USA). The biological evaluation of the products was carried out at the Regional Center for Mycology and Biotechnology at Al-Azhar University, Cairo, Egypt. *Bis*-hydrazonoyl chloride **1** [24] and the methyl arylidene dithiocarbamate **2**, **6**, **8** [25,26,27,28] were prepared as described in the literature.

#### 3.1.2. Synthesis of 1,3,4-Thiadiazoline Derivatives **5a**–**d**, **7a**–**c** and **9a**,**b**

Triethylamine (0.14 mL, 2 mmol) was added dropwise with stirring to a mixture of *bis*-hydrazonoyl chloride **1** (0.451 g, 1 mmol) and the appropriate methyl arylidene dithiocarbamate **2a**–**d**, **6a**–**c** and **8a**,**b** (2 mmol) in ethanol (20 mL) for 30 min. The resulting solid product was collected and recrystallized from DMF to give the corresponding products **5a**–**d**, **7a**–**c** and **9a**,**b**, respectively.

*Diethyl 4,4'-(biphenyl-4,4'-diyl)bis(5-(benzylidenehydrazono)-4,5-dihydro-1,3,4-thiadiazole-2-carboxylate)* (**5a**). Yellow solid, (73% yield), m.p. 194–196 °C; IR (KBr) ν_max_ 3032, 2964 (C-H), 1730 (C=O), 1609 (C=N) cm^−1^; ^1^H-NMR (DMSO-*d*_6_) δ 1.32 (t, 6H, *J* = 7.2 Hz, 2CH_2_CH_3_), 4.35 (q, 4H, *J* = 7.2 Hz, 2CH_2_CH_3_), 7.47–7.88 (m, 14H, Ar-H), 8.04 (d, 4H, *J* = 7.2 Hz, Ar-H); 8.47 (s, 2H, 2CH=N); ^13^C-NMR (DMSO-*d*_6_) δ 13.5(CH_3_), 61.5 (CH_2_), 117.1, 123.4, 126.8, 127.8, 128.1, 129.9, 134.5, 138.2, 142.3, 145.1, 153.6 (Ar-C), 162.5 (C=O). Anal. Calcd. for C_36_H_30_N_8_O_4_S_2_ (702.80): C, 61.52; H, 4.30; N, 15.94. Found: C, 61.38; H, 4.19; N, 15.77%.

*Diethyl 4,4'-(biphenyl-4,4'-diyl)bis(5-((4-methylbenzylidene)hydrazono)-4,5-dihydro-1,3,4-thiadiazole-2-carboxylate)* (**5b**). Yellow solid, (75% yield), m.p. 186–188 °C; IR (KBr) ν_max_ 3049, 2974 (C-H), 1725 (C=O), 1610 (C=N) cm^−1^; ^1^H-NMR (DMSO-*d*_6_) δ 1.29 (t, 6H, *J* = 6.9 Hz, 2CH_2_CH_3_), 2.35 (s, 6H, 2CH_3_), 4.34 (q, 4H, *J* = 6.9 Hz, 2CH_2_CH_3_), 7.21 (d, 4H, *J* = 7.5 Hz, Ar-H), 7.66 (d, 4H, *J* = 7.5 Hz, Ar-H), 7.84 (d, 4H, *J* = 8.7 Hz, Ar-H), 8.05 (d, 4H, *J* = 8.7 Hz, Ar-H), 8.43 (s, 2H, 2CH=N); ^13^C-NMR (DMSO-*d*_6_) δ 14.4, 22.3 (2CH_3_), 61.8 (CH_2_), 115.5, 122.7, 126.9, 127.3, 128.9, 131.2, 139.0, 139.7, 146.5, 147.8, 153.4 (Ar-C), 163.1 (C=O); MS *m*/*z* (%) 730 (M^+^, 4), 627 (9), 457 (26), 226 (41), 119 (100), 87 (73), 59 (69)*.* Anal. Calcd. for C_38_H_34_N_8_O_4_S_2_ (730.86): C, 62.45; H, 4.69; N, 15.33. Found: C, 62.36; H, 4.60; N, 15.24%.

*Diethyl 4,4'-(biphenyl-4,4'-diyl)bis(5-((thiophen-2-ylmethylene)hydrazono)-4,5-dihydro-1,3,4-thiadiazole-2-carboxylate)* (**5c**). Yellow solid, (74% yield), m.p. 251–253 °C; IR (KBr) ν_max_ 3041, 2980 (C-H), 1733 (C=O), 1598 (C=N) cm^−1^; ^1^H-NMR (DMSO-*d*_6_) δ 1.31 (t, 6H, *J* = 6.9 Hz, 2CH_2_CH_3_), 4.34 (q, 4H, *J* = 7.2 Hz, 2CH_2_CH_3_), 7.14–7.73 (m, 6H, Ar-H), 7.84 (d, 4H, *J* = 8.4 Hz, Ar-H), 8.07 (d, 4H, *J* = 8.4 Hz, Ar-H), 8.61 (s, 2H, 2CH=N); ^13^C-NMR (DMSO-*d*_6_) δ 14.4 (CH_3_), 62.7 (CH_2_), 115.5, 123.4, 127.0, 128.0, 130.4, 132.4, 133.3, 139.1, 143.1, 158.3, 159.8 (Ar-C), 165.0 (C=O); MS *m*/*z* (%) 714 (M^+^, 7), 542 (16), 364 (45), 171 (83), 109 (100), 62 (68). Anal. Calcd. for C_32_H_26_N_8_O_4_S_4_ (714.86): C, 53.76; H, 3.67; N, 15.67. Found: C, 53.69; H, 3.50; N, 15.43%.

*Diethyl 4,4'-(biphenyl-4,4'-diyl)bis(5-((furan-2-ylmethylene)hydrazono)-4,5-dihydro-1,3,4-thiadiazole-2-carboxylate)* (**5d**). Yellow solid, (70% yield), m.p. 283–285 °C; IR (KBr) ν_max_ 3050, 2973 (C-H), 1732 (C=O), 1616 (C=N) cm^−1^; ^1^H-NMR (DMSO-*d*_6_) δ 1.32 (t, 6H, *J* = 7.2 Hz, 2CH_2_CH_3_), 4.35 (q, 4H, *J* = 7.2 Hz, 2CH_2_CH_3_), 7.08–7.71 (m, 6H, Ar-H), 7.87 (d, 4H, *J* = 8.4 Hz, Ar-H), 8.05 (d, 4H, *J* = 8.4 Hz, Ar-H), 8.62 (s, 2H, 2CH=N); MS *m*/*z* (%) 682 (M^+^, 3), 368 (16), 224 (29), 152 (35), 80 (100), 52 (84). Anal. Calcd. for C_32_H_26_N_8_O_6_S_2_ (682.73): C, 56.30; H, 3.84; N, 16.41. Found: C, 56.17; H, 3.81; N, 16.28%.

*Diethyl 4,4'-(biphenyl-4,4'-diyl)bis(5-((1-(naphthalen-2-yl)ethylidene)hydrazono)-4,5-dihydro- 1,3,4-thiadiazole-2-carboxylate)* (**7a**). Yellow solid, (72% yield), m.p. 243–245 °C; IR (KBr) ν_max_ 3046, 2924 (C-H), 1718 (C=O), 1599 (C=N) cm^−1^; ^1^H-NMR (DMSO-*d*_6_) δ 1.32 (t, 6H, *J* = 7.2 Hz, 2CH_2_CH_3_), 2.49 (s, 6H, 2CH_3_), 4.35 (q, 4H, *J* = 7.2 Hz, 2CH_2_CH_3_), 7.46–8.19 (m, 20H, Ar-H), 8.34 (s, 2H, Naphthalene-H1); MS *m*/*z* (%) 830 (M^+^, 17), 378 (88), 263 (29), 177 (48), 127 (100), 63 (71). Anal. Calcd. for C_46_H_38_N_8_O_4_S_2_ (830.98): C, 66.49; H, 4.61; N, 13.48. Found: C, 66.36; H, 4.49; N, 13.41%.

*Diethyl 4,4'-(biphenyl-4,4'-diyl)bis(5-((1-(furan-2-yl)ethylidene)hydrazono)-4,5-dihydro-1,3,4-thiadiazole-2-carboxylate)* (**7b**). Yellow solid, (73% yield), m.p. 259–261 °C; IR (KBr) ν_max_ 3055, 2978, 2928 (C-H), 1715 (C=O), 1601 (C=N) cm^−1^; ^1^H-NMR (DMSO-*d*_6_) δ 1.34 (t, 6H, *J* = 7.2 Hz, 2CH_2_CH_3_), 2.32 (s, 6H, 2CH_3_), 4.35 (q, 4H, *J* = 7.2 Hz, 2CH_2_CH_3_), 6.63 (d, 2H, *J* = 2.7 Hz), 7.02 (d, 2H, *J* = 2.7 Hz), 7.39 (m, 2H), 7.87 (d, 4H, *J* = 9.0 Hz, Ar-H), 8.05 (d, 4H, *J* = 9.0 Hz, Ar-H); ^13^C-NMR (DMSO-*d*_6_) δ 14.4, 15.1(2CH_3_), 62.3 (CH_2_), 116.5, 122.6, 127.9, 128.3, 128.9, 133.2, 138.0, 142.7, 146.7, 153.0, 157.8 (Ar-C), 163.4(C=O); MS *m/z* (%) 710 (M^+^, 20), 447 (22), 226 (29), 109 (52), 59 (100). Anal. Calcd. for C_34_H_30_N_8_O_6_S_2_ (710.78): C, 57.45; H, 4.25; N, 15.76. Found: C, 57.32; H, 4.21; N, 15.53%.

*Diethyl 4,4'-(biphenyl-4,4'-diyl)bis(5-((1-(pyridin-3-yl)ethylidene)hydrazono)-4,5-dihydro-1,3,4-thiadiazole-2-carboxylate)* (**7c**). Yellow solid, (70% yield), m.p. 263–265 °C; IR (KBr) ν_max_ 3059, 2922 (C-H), 1736 (C=O), 1599 (C=N) cm^−1^; ^1^H-NMR (DMSO-*d*_6_) δ 1.31 (t, 6H, *J* = 7.2 Hz, 2CH_2_CH_3_), 2.49 (s, 6H, 2CH_3_), 4.32 (q, 4H, *J* = 7.2 Hz, 2CH_2_CH_3_), 7.48–7.75 (m, 2H, Pyridine-H), 7.84 (d, 4H, *J* = 8.4 Hz, Ar-H), 8.07 (d, 4H, *J* = 8.4 Hz, Ar-H), 8.29–9.05 (m, 6H, Pyridine-H); ^13^C-NMR (DMSO-*d*_6_) δ 14.6, 15.3 (2CH_3_), 62.6 (CH_2_), 114.2, 115.5, 120.5, 127.3, 128.0, 130.8, 132.2, 134.2, 142.1, 143.5, 152.6, 159.8 (Ar-C), 167.1 (C=O); MS *m/z* (%): 732 (M^+^, 3), 415 (24), 276 (37), 205 (64), 152 (60), 84 (100). Anal. Calcd. for C_36_H_32_N_10_O_4_S_2_ (732.83): C, 59.00; H, 4.40; N, 19.11 Found: C, 58.86; H, 4.31; N, 19.02%.

*Diethyl 4,4'-(biphenyl-4,4'-diyl)bis(5-(cyclohexylidenehydrazono)-4,5-dihydro-1,3,4-thiadiazole-2-carboxylate)* (**9a**). Yellow solid, (72% yield), m.p. 233–235 °C; IR (KBr) ν_max_ 3032, 2929 (C-H), 1716 (C=O), 1616 (C=N) cm^−1^; ^1^H-NMR (DMSO-*d*_6_) δ 1.34 (t, 6H, *J* = 7.2 Hz, 2CH_2_CH_3_), 1.61 (m, 12H, 6CH_2_), 2.34 (m, 4H, 2CH_2_), 2.63 (m, 4H, 2CH_2_), 4.38 (q, 4H, *J* = 7.2 Hz, 2CH_2_CH_3_), 7.88 (d, 4H, *J* = 8.4 Hz, Ar-H), 8.04 (d, 4H, *J* = 8.4 Hz, Ar-H); ^13^C-NMR (DMSO-*d*_6_) δ 14.4 (CH_3_), 25.0, 27.5, 35.2, 63.4 (CH_2_), 116.1, 122.6, 126.2, 133.6, 137.2, 146.5, 160.0 (Ar-C), 163.1(C=O); MS *m*/*z* (%): 686 (M^+^, 6), 415 (16), 226 (24), 194 (52), 104 (79), 90 (100). Anal. Calcd. for C_34_H_38_N_8_O_4_S_2_ (686.85): C, 59.45; H, 5.58; N, 16.31. Found: C, 59.25; H, 5.39; N, 16.22%.

*Ethyl 4-(4'-((Z)-5-(ethoxycarbonyl)-2-((2-oxoindolin-3-ylidene)hydrazono)-1,3,4-thiadiazol-3(2H)-yl)biphenyl-4-yl)-5-((Z)-(2-oxoindolin-3-ylidene)hydrazono)-4,5-dihydro-1,3,4-thiadiazole-2-carboxylate* (**9b**). Yellow solid, (67% yield), m.p. 266–268 °C; IR (KBr) ν_max_ 3344 (NH), 3033, 2978 (C-H), 1724, 1681 (2C=O), 1607 (C=N) cm^−1^; ^1^H-NMR (DMSO-*d*_6_) δ 1.34 (t, 6H, *J* = 6.9 Hz, 2CH_2_CH_3_), 4.42 (q, 4H, *J* = 6.9 Hz, 2CH_2_CH_3_), 6.85–8.01 (m, 16H, Ar-H), 10.65 (s, br, 2H, 2NH, D_2_O-exchangeable); ^13^C-NMR (DMSO-*d*_6_) δ 14.3 (CH_3_), 62.6 (CH_2_), 114.2, 115.5, 117.5, 120.5, 123.5, 124.1, 126.9, 127.3, 130.8, 131.2, 134.2, 142.1,159.8 (Ar-C), 163.4, 170.6 (2C=O); MS *m*/*z* (%): 784 (M^+^, 4), 599 (18), 392 (26), 257 (38), 166 (68), 70 (100), 58 (42). Anal. Calcd. for C_38_H_28_N_10_O_6_S_2_ (784.82): C, 58.15; H, 3.60; N, 17.85. Found: C, 58.08; H, 3.47; N, 17.69%.

### 3.2. Pharmacology

#### Anticancer Activity

The cytotoxic evaluation of the synthesized compounds was carried out at the Regional Center for Mycology and Biotechnology at Al-Azhar University, Cairo, Egypt according to the reported method [29].

## 4. Conclusions

Various *bis*(1,3,4-thiadiazole) derivatives were synthesized in efficient and easy protocol. Biological studies revealed that *bis*-thiadiazole derivatives **5c**,**d**, **7b**,**c** and **9b** had higher antitumor activity than the standard drug Imitanib.

## Figures and Tables

**Table 1:** The antitumor activities of the tested *bis*-thiadiazoles **5a**–**d**, **7a**–**c**, **9a**,**b**.

Compd. No.	R	IC_50_ (µg/mL)
**5a**	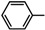	45.2 ± 1.4
**5b**	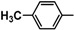	37.5 ± 1.7
**5c**	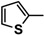	18.4 ± 1.2
**5d**	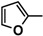	20.2 ± 0.6
**7a**	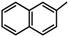	145.1 ± 3.4
**7b**	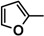	21.7 ± 1.5
**7c**	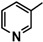	16.9 ± 1.7
**9a**	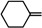	80.0 ± 2.0
**9b**	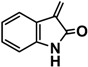	20.7 ± 2.4
**Doxorubicin**	----	0.8 ± 0.1
**Imatinib**	----	24.5 ± 0.3
